# Research on the generation and annotation method of thin section images of tight oil reservoir based on deep learning

**DOI:** 10.1038/s41598-024-63430-z

**Published:** 2024-06-04

**Authors:** Tao Liu, Zongbao Liu, Kejia Zhang, Chunsheng Li, Yan Zhang, Zihao Mu, Mengning Mu, Mengting Xu, Yue Zhang, Xue Li

**Affiliations:** 1 School of Computer and Information Technology, Northeast Petroleum University, Daqing, 163318 China; 2 School of Earth Sciences, Northeast Petroleum University, Daqing, 163318 China; 3 Exploration and Development Research Institute of Huabei Oilfield Company, Renqiu, 062552 China

**Keywords:** Tight oil reservoir, Cast thin section image, Deep learning, Image generation, Reservoir evaluation, Petrology, Energy storage, Mathematics and computing

## Abstract

The cast thin sections of tight oil reservoirs contain important parameters such as rock mineral composition and content, porosity, permeability and stratigraphic characteristics, which are of great significance for reservoir evaluation. The use of deep learning technology for intelligent identification of thin section images is a development trend of mineral identification. However, the difficulty of making cast thin sections, the complexity of the making process and the high cost of thin section annotation have led to a lack of cast thin section images, which cannot meet the training requirements of deep learning image recognition models. In order to increase the sample size and improve the training effect of deep learning model, we proposed a generation and annotation method of thin section images of tight oil reservoir based on deep learning, by taking Fuyu reservoir in Sanzhao Sag as the target area. Firstly, the Augmentor strategy space was used to preliminarily augment the original images while preserving the original image features to meet the requirements of the model. Secondly, the category attention mechanism was added to the original StyleGAN network to avoid the influence of the uneven number of components in thin sections on the quality of the generated images. Then, the SALM annotation module was designed to achieve semi-automatic annotation of the generated images. Finally, experiments on image sharpness, distortion, standard accuracy and annotation efficiency were designed to verify the advantages of the method in image quality and annotation efficiency.

## Introduction

Cast thin sections is a kind of rock thin section made by injecting colored liquid gel into the rock pore space under vacuum pressure, and grinding the liquid gel into thin section after curing, which can reflect the information of formation properties, rock types, pore structures, permeability, etc., which are of great significance for reservoir evaluation of oilfields. Since rock samples fracture easily during rock coring, and the making process of thin section samples is complex and expensive, thin section samples and thin section image data are scarce, and cannot meet the demand of intelligent algorithms for sample quantity. In addition, intelligent algorithms require geological experts to annotate images as input, so thin section annotation and identification still rely on geological experts, wasting the time and energy of geological experts. Currently, researchers typically use data transformation methods or data oversampling methods to augment images, and generate “new images” by transforming the original images, so that the model can extract more useful information, thus improving the generalization ability of the model^1^.

Data transformation methods operate on a single image, and change the expression form of the original image through various transformation operations (rotation, scaling, distortion, etc.), quickly generating a large number of “new images” different from the original image^2^. Although these methods are simple and easy to implement, they only use prior knowledge of the image itself when generating new images, leading to a lack of data diversity and limited accuracy improvement that the model can achieve in practical applications.

Generative Adversarial Networks (GANs) are a representative technology in data oversampling methods^3^. Through oversampling in data distribution, “new images” that do not exist in real scenes, effectively increase data diversity. However, this network requires a large amount of data for training and is difficult to generate high-resolution images. Tero Karras et al.^4–6^ studied the application of GANs in generating high-resolution images, and effectively improved the quality of the generated images under small-scale data training by redesigning the structure of the generator and adding adaptive discriminator augmentation. However, in the case of large number of image foreground objects, complex structure and relatively small size of training data, the quality of the generated images is not ideal^7^.

Based on the above analysis, it is difficult to solve the problems of limited quantity, complex image structure, difficult annotation, etc. in the augmentation of thin section images of tight oil by using data transformation methods or data oversampling methods alone. Therefore, we integrate data transformation methods and data oversampling methods, combine them with a semi-automatic annotation module, and propose a thin section image generation method based on Semi-Automatic Labeling of Categories Attention StyleGAN (SACA-StyleGAN); The annotation of generated image samples is completed on the basis of image augmentation.

## SACA-StyleGAN method

### Algorithm framework

**Figure 1:**
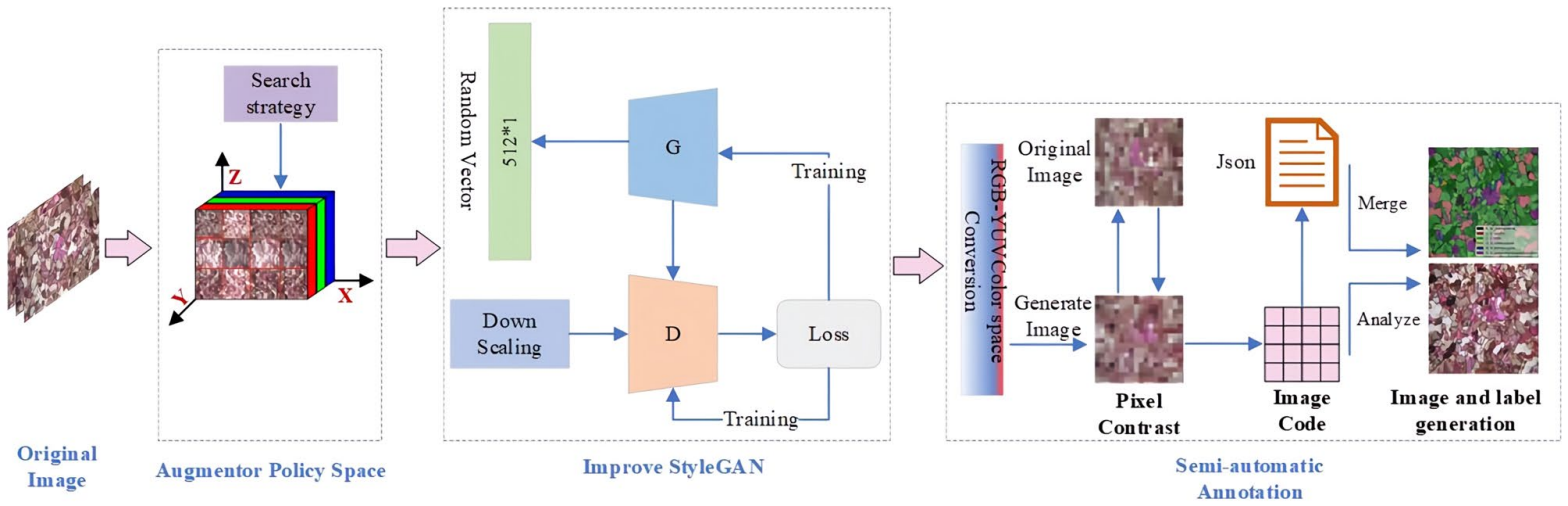
SACA-StyleGAN method process.

The SACA-StyleGAN algorithm consists of three parts: data construction, image generation and semi-automatic annotation module, as shown in Fig. 1. Data construction adopts the Augmentor method to preliminarily augment the original image dataset to meet the data quantity requirements of the original StyleGAN network^4^. Image generation uses the category attention mechanism to improve the original StyleGAN network (CA-StyleGAN), avoiding the influence of uneven components of thin section images on the generated images and improving the quality of the generated images. The semi-automatic annotation module (The "SA" part of SACA-StyleGAN) completes the semi-automatic annotation of thin section images through color space conversion, pixel comparison, image encoding, image data fusion and analysis, reducing the working pressure of geological personnel to annotate thin section images to some extent.

### Dataset construction

The dataset construction is based on cast thin section images annotated under the guidance of geological experts. An Augment strategy space and its strategy search algorithm were designed to complete the dataset construction. The Labelme tool was used for semantic annotation of the original image to ensure the accuracy of the label. The labels consist of seven types: Quartz, Feldspar, Lithic, Primary Pore (PP), Casting Pore (CP), Cemented Dissolution Pore (CDP) and Microcrack.

The Augment strategy space is a three-dimensional space that stores all possible image augmentation operations. The X-axis represents the strategy name, the Y-axis represents the strategy type, and the Z-axis represents the parameter range. The specific strategy is shown in Table 1.

**Table 1:** Augment strategy space.

Strategy name	Strategy weighting (%)	Strategy type	Type weight (%)	Parameter range
Spatial Scaling (Maintaining component ratios)	20	Enlarge/Shrink	50	[0.8, 1.2]
Rotate	10	Clockwise/Anticlockwise	50	[0, 90]
Cut	15	Level/Vertical	50	[− 15, 15]
Flip	10	Level/Vertical /Diagonal	33	
Luminance	10	Weaken/Enhance	50	[0.7, 1.3]
Contrast	15	Weaken /Enhance	50	[0.7, 1.3]
Elastic deformation	20	X-axis/Y-axis /XY-axis	33	[2, 8]

**Figure 2:**
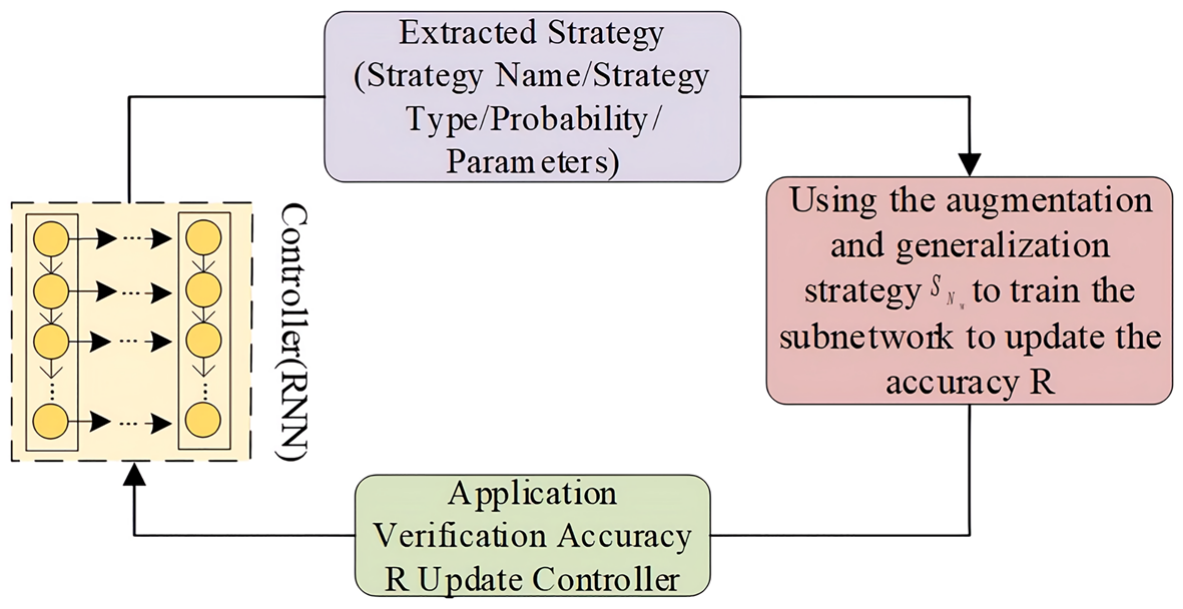
Search strategy for selecting image augmentation operations from Augment strategy space.

In the data construction process, different augmentation methods and parameter combinations were selected through strategy search to form an amplification strategy. To avoid the excessive influence of a single operation on the image or the loss of original image features of the generated images due to multiple operations, constraints were set such that each strategy can only be executed once and each augmentation can involve at most two strategies. The goal of the strategy search is to quickly search for the selection and sequence of amplification strategy in the strategy space to better suit GAN for the constructed dataset. The search strategy includes a recurrent neural network (single-layer LSTM) as a controller and a training algorithm (gradient descent method)^8^. The specific process is illustrated in Fig. 2.

The optimization problem to be solved for the search strategy problem can be defined by Eq. (1).

1 $$\left\{ \begin{aligned} & w_{{}}^{ * } (s) = \begin{array}{*{20}c} {\arg \min } \\ w \\ \end{array} f_{train} (w,s) \\ & \begin{array}{*{20}c} {\min } \\ s \\ \end{array} f_{val} (w_{{}}^{ * } (s),s) \\ \end{aligned} \right.$$

where $f(w,s)$ represents the differentiable objective function. $s \in S$ represents the amplification strategy. $w \in W$ represents the network parameter. $f_{train}$ represents the training loss. $f_{val}$ represents the validation loss. Therefore, this section converts (transforms) the strategy search into that of gradient-based optimization of the differentiable objective function $f(w,s)$ with respect to network parameter $w$ and amplification strategies $s$. Since the gradient $\nabla_{s} f$ of the amplification strategy $s$ cannot be directly obtained, the stochastic loose strategy^9^ is introduced to transform the coupling optimization problem of strategy and strategic weight manipulation into a differentiable objective optimization problem of the objective function, and the natural gradient descent strategy^10^ is applied to complete the strategy and weight optimization. The stochastic loose strategy replaces the gradient $\nabla_{s} f$ of the amplification strategy by defining the probability distribution $p_{\theta } (s)$ with $\theta$ as the parameter, aiming to minimize the validation loss $f_{val}$ of the objective function $f(w,c)$ with respect to parameter $\theta$.

2 $$\left\{ \begin{aligned} & \begin{array}{*{20}c} {\min } \\ \theta \\ \end{array} J(w,\theta ) = \int {_{s \in S} } f_{val} (w_{{}}^{ * } (s),s)p_{\theta } (s)ds \\ & w_{{}}^{ * } (s) = \begin{array}{*{20}c} {\arg \min } \\ w \\ \end{array} f_{train} (w,s) \\ \end{aligned} \right.$$

where $J$ is the stochastic loose objective function, with all attributes of $f(w,c)$, and makes $w$ and $\theta$ differentiable. To simplify the process of updating $w_{{}}^{t}$ and $\theta_{{}}^{t}$, Monte-Carlo (MC) is applied to estimate the gradient $\nabla_{w} J(w_{{}}^{t} ,s_{i} )$ of $w$ and the gradient $\nabla_{\theta } J(w,\theta )$ of $\theta$. Simultaneously, the adaptive stochastic natural gradient descent is used for updating $w_{{}}^{t}$, and the natural gradient descent is employed for updating $\theta_{{}}^{t}$.

3 $$G_{w} (w_{{}}^{t} ,\theta_{{}}^{t} ) = \frac{1}{{N_{w} }}\sum\limits_{i = 1}^{{N_{w} }} {\nabla_{w} } f_{train} (w_{{}}^{t} ,s_{i} )$$

4 $$w_{{}}^{t + 1} = w_{{}}^{t} - l_{w} G_{w} (w_{{}}^{t} ,\theta_{{}}^{t} )$$

5 $$\theta_{{}}^{t + 1} = \theta_{{}}^{t} - l_{\theta } F(\theta^{t} )_{{}}^{ - 1} \frac{1}{{N_{\theta } }}\sum\limits_{j = 1}^{{N_{\theta } }} {\nabla_{\theta } } f_{val} (w_{{}}^{t + 1} ,s_{j} )\ln p_{\theta } (s_{j} )$$

where $G_{w} (w_{{}}^{t} ,\theta_{{}}^{t} )$ represents the gradient $\nabla_{w} J(w_{{}}^{t} ,s_{i} )$ of $w$ estimated by Monte-Carlo (MC); $l_{\theta }$ denotes the learning rate; and $F(\theta_{t} )$ indicates the Fisher matrix, with the same calculation process^9^.

### CA-StyleGAN thin section image generation algorithm

**Figure 3:**
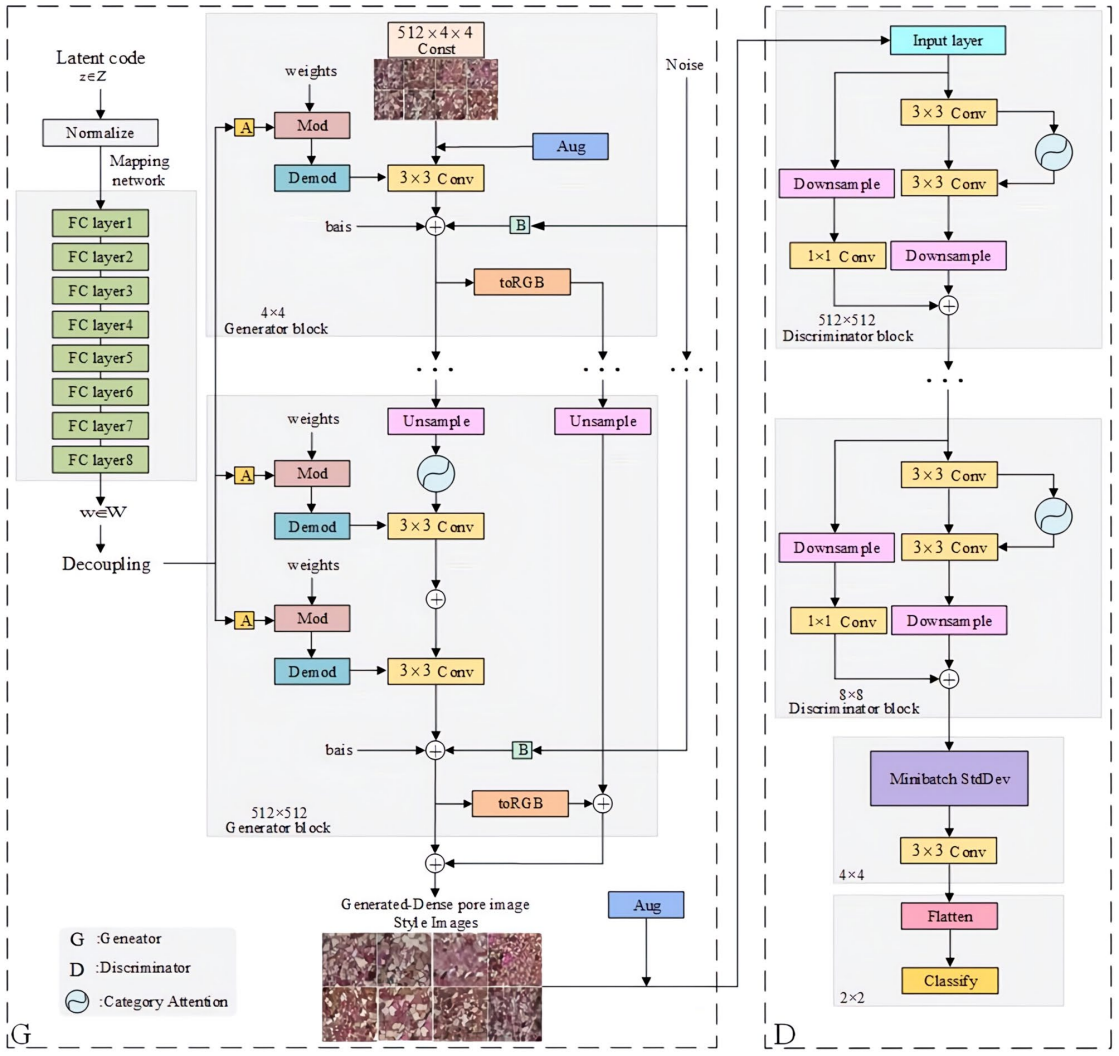
CA-StyleGAN network architecture.

The original StyleGAN achieves natural image generation through latent code, noise and discriminator^11^. Compared to natural images, tight oil thin section images exhibit features such as numerous fine particles, underdeveloped pores and data imbalance. In order to improve the generation quality of thin section images, in this section, the Style control method was modified based on the original StyleGAN network, and the category attention mechanism was introduced to enhance the generator’s attention to the associated information of pore components, texture features and congruent pixel. Moreover, the noise input was reduced regarding the characteristics of darker tone and lower resolution of the cast thin section of Fuyu reservoir in Sanzhao Sag, and the Two Time-Scale Update Rule (TTUR) was employed to optimize the training process^12^. The improved model was named CA-StyleGAN, and its network structure is shown in Fig. 3. The CA-StyleGAN method will be elaborated in detail below.

1. Replace mixing regularization with latent code. There are numerous mineral components in the thin section images of Fuyu reservoir in Sanzhao Sag. Direct application of latent codes and mixing regularization of the original StyleGAN network can lead to the blending of features of different mineral components, which will result in mineral adhesion in the generated image, making it indistinguishable. To this end, the latent code was applied instead of mixing regularization to control the shape, texture and color generation of each mineral in the thin section image to avoid the distortion, overlapping, indistinguishable problems of minerals.
2. Modify the noise input. In view of the similar characteristics of each particle and various pore types in the cast thin section image, and the characteristics of a single color, the generator structure was simplified, the resolution of the final module was changed from the original 1024 × 1024 to 512 × 512, the number of network parameters was reduced while ensuring image generation quality, to improve the generator efficiency. Additionally, the noise input for each network of the generator was modified once to avoid introducing excessive noise that may affect image quality. The generation method of noise $N$ can be represented by Eq. (6).
  6 $$N\left( {\omega ,\chi } \right) = randn\left( {H,W} \right) \times \omega + \chi ,\omega \in R^{channel} ,\chi \in R^{channel \times H \times W}$$

where $N\left( {\omega ,\chi } \right)$ represents the noise function, input $\omega$ and $\chi$ two parameters, output the modified noise. $\omega$ is the noise scaling factor, which is used to adjust the amplitude of the generated random number, and $\chi$ is the noise offset. $randn\left( {H,W} \right)$ is used to generate a matrix of standard normally distributed random numbers of size $H \times W$, where $H$ and $W$ are 512 pixels.

**Figure 4:**
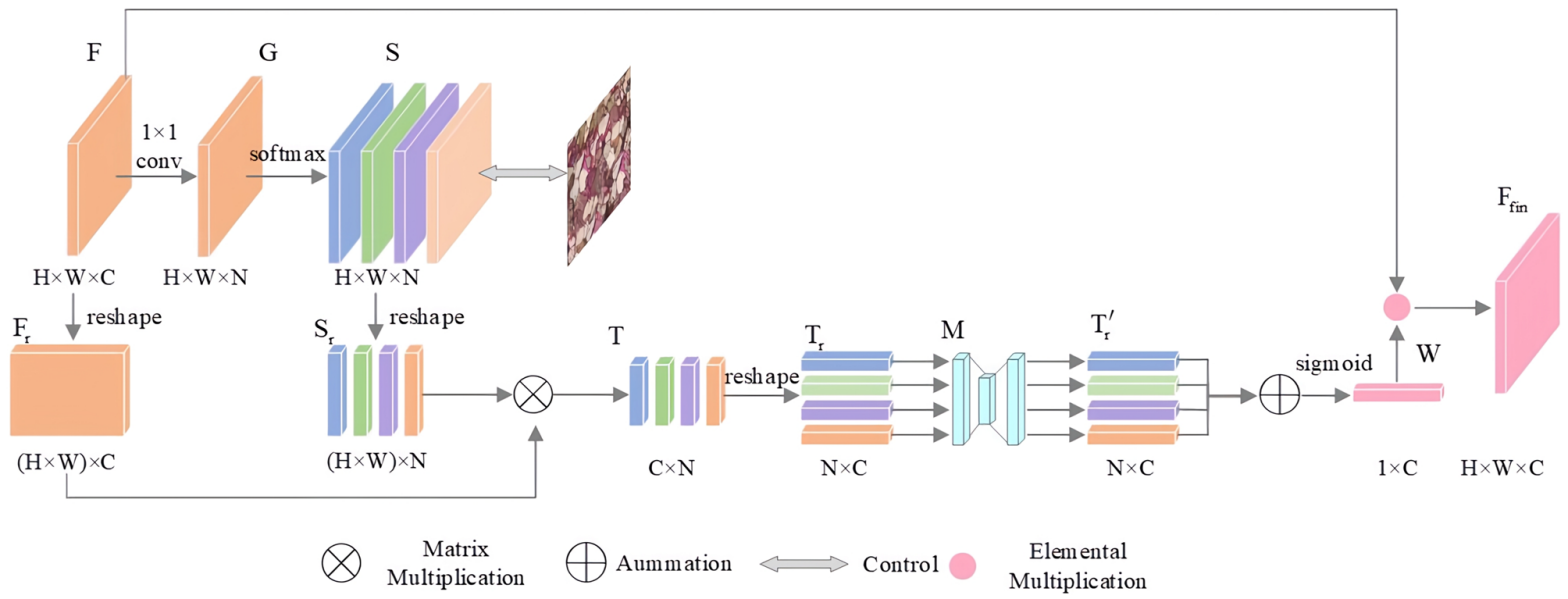
Class attention mechanism.

(3) Add the category attention mechanism. The training process of StyleGAN relies on the convolutional kernel to capture the receptive field, which cannot help obtain global information of thin section images and can easily cause long-distance pixel information loss^13^. Moreover, the severe imbalance in the number of pores and particle components in thin section images results in insufficient learning of pore features, affecting image quality. Therefore, the category attention mechanism was added to the generator and discriminator networks to learn long-distance pixel correlation features and pore features in thin section images, and enhance the discriminator capability. The category attention mechanism strengthens pore category features through five steps: feature classification, dimension adjustment, multi-layer perceptron, function normalization and feature layer output. The structure is shown in Fig. 4.

Feature classification classifies the input feature map $F \in R^{H \times W \times C}$ into the class map $S \in R^{H \times W \times N}$, and the process can be defined by Eq. (7). Where, $f^{1 \times 1}$ represents the convolution size, and ${\text{softmax}}()$ represents the function.

7 $$S = soft\max \left( {f^{1 \times 1} \left( F \right)} \right)$$

Dimension adjustment includes the expansion of the feature map $F$ and the class map $S$ in width and height dimensions, as well as the matrix operation after expansion. This process can be defined by Eqs. (8), (9) and (10). Where, $F_{{\text{r}}}$ represents the matrix obtained after expansion and adjustment of feature $F$ in width and height dimensions; $S_{{\text{r}}}$ represents the matrix obtained by expanding the class map $S$ in width and height dimensions; $reshape$ denotes dimension adjustment; $T_{{\text{r}}}$ represents the matrix obtained by multiplying matrices $F_{{\text{r}}}$ and $S_{{\text{r}}}$, showing the correlation between feature channels and pore classes, and the value obtained is proportional to the correlation; $\otimes$ represents matrix multiplication.

8 $$F_{{\text{r}}} = reshape\left( F \right),F_{{\text{r}}} \in R^{{\left( {H \times W} \right) \times C}}$$

9 $$S_{{\text{r}}} = reshape\left( S \right),F_{{\text{r}}} \in R^{{\left( {H \times W} \right) \times N}}$$

10 $$T_{{\text{r}}} = reshape\left( {F_{{\text{r}}} \otimes S_{{\text{r}}} } \right)$$

The role of the multi-layer perceptron is to transform $T_{{\text{r}}}$ into the attention map $T^{\prime}_{r} \in R^{N \times C}$ and sum each line in $T^{\prime}_{r}$.

Function normalization applies the Sigmoid function to normalize the sum of each line of $T^{\prime}_{r}$, obtaining the channel attention weight $W$, which can be defined by Eq. (11). Where, $i$ represents the i-th line in $T_{{\text{r}}}$; $MLP$ represents the multi-layer perceptron; $N$ represents the class number; $\sigma$ represents the Sigmoid function.

11 $$W = \sigma \left( {\sum\limits_{i = 1}^{N} {MLP\left( {T_{r}^{i} } \right)} } \right),i \in \left\{ {1,2, \cdots ,N} \right\}$$

The role of the feature layer output is to multiply the feature map $F$ with the channel attention weight $W$ element by element to obtain the output feature map $F_{fin}$. $F_{fin}$ contains two types of related information: feature channel and category, and strengthens the relevant features of pore category, which can be defined by Eq. (12). Where, $\odot$ represents element-by-element multiplication.

12 $$F_{fin} = F \odot W$$

(4) TTUR. To balance the training speed of the generator and discriminator, and accelerate the convergence of the discriminator, the TTUR is adopted to automatically set different learning rates for training the generator and discriminato^14^. The model training is based on the stochastic gradient $\tilde{g}\left( {\theta ,\omega } \right)$ of the generator loss function $L_{G}$ and the stochastic gradient ${\tilde{\text{d}}}\left( {\theta ,\omega } \right)$ of the discriminator loss function $L_{D}$. Where, $\theta$ and $\omega$ represent the learning variable parameters of the generator and discriminator, respectively. The training process involves randomly selecting m samples from both the thin section image sample $P_{D}$ and generated image sample $P_{G}$ to form real sample ${\text{x}}^{(i)}$ and generated sample $z^{(i)}$, $1 \le {\text{i}} \le m$. If the actual gradients of the generator and discriminator are $g\left( {\theta ,\omega } \right) = \nabla_{\theta } L_{G}$ and $d\left( {\theta ,\omega } \right) = \nabla_{\omega } L_{D}$, $\tilde{g}\left( {\theta ,\omega } \right)$ and ${\tilde{\text{d}}}\left( {\theta ,\omega } \right)$ are redefined using stochastic variables $M^{\left( \theta \right)}$ and $M^{\left( \omega \right)}$ (Eqs. 13 and 14). At this point, $\tilde{g}\left( {\theta ,\omega } \right)$ and ${\tilde{\text{d}}}\left( {\theta ,\omega } \right)$ are approximate to the actual gradient. The gradient process of learning parameters for the generator and discriminator can be defined by Eqs. (15) and (16). Where, $a\left( n \right)$ represents the generator learning rate, and $b\left( n \right)$ represents the discriminator learning rate.

13 $$\tilde{g}\left( {\theta ,\omega } \right) = g\left( {\theta ,\omega } \right) + M^{\left( \theta \right)}$$

14 $$\tilde{d}\left( {\theta ,\omega } \right) = h\left( {\theta ,\omega } \right) + M^{\left( \omega \right)}$$

15 $$\theta_{n + 1} = \theta_{n} + a\left( n \right) \times \left( {g\left( {\theta_{n} ,\omega_{n} } \right) + M_{n}^{\left( \theta \right)} } \right)$$

16 $$\omega_{n + 1} = \omega_{n} + b\left( n \right) \times \left( {d\left( {\omega_{n} ,\omega_{n} } \right) + M_{n}^{\left( \omega \right)} } \right)$$

### SALM annotation module design

Based on the related research ideas of petrophysical models, the Labelme labeling principle is analyzed, and the semi-automatic labeling module (SALM) is designed to complete the preliminary labeling of each component of thin section images^15,16^. SALM shifts the focus of geological experts from original “annotation” to “quality detection”, reduces workload, saves the labor cost, and achieves generation and semi-automatic annotation of thin section images. Based on a small number of manual annotation results, the SALM annotation module converts the RGB pixel values of various components in thin section images to YUV color description through the color space conversion mechanism, and calculates the main distribution interval of each component Y, U and V based on the mean value of single component Y, U and V to provide a basis for the edge judgment of new images. By traversing the newly generated thin section images, the Y, U and V components of each pixel are obtained and compared with the main distribution interval respectively to determine the edge position coordinates of each component. The image coding mechanism was designed to convert the cast thin section image into serialized data, and generate the Json annotation information file; then the Json file generated by the thin section image is fused with the image, to complete the image data fusion; Finally, the semi-automatic image annotation is completed by parsing the Json annotation information file. The details are described below.

#### Color space conversion

Geological experts mainly annotate cast thin section images by comprehensively analyzing the optical characteristics such as luminance and color of each component of the thin section. In the color space, only the YUV color space can analyze image fractal features and represent the hue through luminance (Y) and chroma (UV), i.e. hue and saturation^17^. The YUV color space shares the same basis as geological experts annotating the components of thin section images, so the thin section image is converted from RGB color space to YUV color space to reflect the color boundary of each component more accurately. The equation for conversion of RGB color space to YUV color space by Gamma calibration is shown below.

17 $$\left\{ \begin{gathered} Y = 0.299R + 0.587G + 0.114B \hfill \\ U = - 0.147R - 0.289G + 0.436B \hfill \\ V = 0.615R - 0.515G - 0.100B \hfill \\ \end{gathered} \right.$$

#### Pixel comparison

Pixel comparison includes two parts: pixel statistics and pixel traversal for each component of the labeled image. The pixel of each component in the labeled image calculates the mean values of Y, U and V components of each component of the original cast thin section image annotated by geological experts through the PIL algorithm, and counts the main distribution intervals of Y, U and V components of each component, to provide a basis for dividing the edges of each component of the generated image. Pixel traversal reads and traverses the generated image by the PIL algorithm, compares each pixel of the new image with the distribution intervals of Y, U, V components of each component, determines the component type of the pixel, and then records the coordinates of each connected domain to provide component position information for generating the Json annotation information file.

#### Image coding

Image coding uses the base64 algorithm for utf-8 coding of the generated images, establishes the information dictionary containing the original byte codes of the coordinates of each component of the thin section, and then applies the json algorithm to generate the Json annotation information file from the information dictionary.

#### Image data fusion and analysis

Image data fusion imitates the labeling mechanism of labelme software, traverses the coordinates of the edges of each component and their corresponding image data through the fusion method, the shapes algorithm to construct shape information of each component in the generated image, including coordinate points, labels, fill colors and line colors, then the json algorithm is used to combine the image path, image data, object shape, etc., to realize the fusion of coordinates and corresponding images, generate the final JSON information file, and analyze it to complete the semi-automatic annotation of the cast thin section image.

## Experimental design

### Experimental introduction

All experiments in this section were conducted on the Ubuntu 20.04.3 operating system, with Intel Xeon Silver 4210R CPU running at 2.4GHz; 24GB GPU memory, 64GB RAM, NVIDIA Quadro RTX 6000 model; Pytorch 1.8.0 environment; and Cuda11.2 computing platform.

The experiments include image sharpness detection, image distortion detection and annotation accuracy detection. Image sharpness detection and image distortion detection are for evaluating image quality, while annotation accuracy detection is for evaluating the annotation effect. The image sharpness detection experiment compares CA-StyleGAN with similar image generation algorithms in terms of sharpness and diversity of generated images to verify the advantages of CA-StyleGAN in sharpness and diversity of generated images. The image distortion detection experiment compares the distortion degree of images generated by CA-StyleGAN with similar image generation algorithms to demonstrate the authenticity of images generated by CA-StyleGAN. The annotation accuracy detection experiment compares SALM with manual annotation results to demonstrate the annotation effect of SALM. The annotation efficiency detection experiment analyzes the time required for annotating different numbers of thin section images using SALM to verify the annotation efficiency and stability of SALM under different datasets.

### Experimental design

#### Image sharpness detection experiment design

The image sharpness detection experiment uses Inception Score (IS), Frechet Inception Distance (FID), and Kernel Inception Distance (KID) as evaluation indexes^18–20^.

IS places the newly generated thin section images into the Inception Net-V3 image classification network and calculates the entropy of each component of the thin section to determine the sharpness and diversity of thin section images. When measuring sharpness, a smaller entropy value indicates clearer components in the thin section images, and when measuring diversity, a larger entropy value indicates higher diversity. The IS calculation method can be represented by Eq. (18).

18 $$IS\left( g \right) = \exp \left( {E_{{x\sim P_{g} }} D_{KL} \left( {{\text{p}}\left( {y|x} \right)||{\text{p}}\left( y \right)} \right)} \right)$$

where $\exp$ is to facilitate comparison of the final calculated IS values, has no specific meaning; $E_{{x\sim P_{g} }}$ is the expected value operator; $x\sim P_{g}$ represents the sample distribution of each component of the generated thin section image; ${\text{p}}\left( {y|x} \right)$ represents the probability distribution that each component $x$ of the generated thin section image belongs to all categories $y$; ${\text{p}}\left( y \right)$ represents the marginal distribution; $D_{KL}$ represents the KL divergence measures the distance between two probability distributions.

FID obtains n-dimensional feature vectors of thin section image samples through the Inception Network and applies the Frechet distance to calculate the distribution distance between the feature vectors of generated thin section images and original thin section images, as the basis for determining the authenticity of generated thin section image samples. Therefore, a smaller FID value indicates a higher similarity between the generated thin section images and the original thin section image samples. The FID calculation method can be represented by Eq. (19)

19 $$FID = ||\mu_{{\text{r}}} - \mu_{g} ||^{2} + Tr\left( {\sum\nolimits_{r} { + \sum\nolimits_{g} { - 2\left( {\sum {_{r} \sum {_{g} } } } \right)^{1/2} } } } \right)$$

where $\mu_{{\text{r}}}$ represents the mean of the original thin section image features; $\mu_{g}$ represents the mean of the generated thin section image features; $\sum {_{r} }$ represents the covariance matrix of the original thin section image features; $\sum {_{g} }$ represents the covariance matrix of the generated thin section image features; $Tr$ represents the trace of matrix; $|| \cdot ||^{2}$ represents the Euclidean distance norm. Since FID does not need to classify the generated thin section image samples, it can effectively avoid the impact of the fuzzy category of generated thin section image samples and ImageNet dataset classification on the calculation results.

Similar to the principle of FID, KID extracts feature vectors of original thin section images and generated thin section images through the Inception Network, calculates the square of the maximum mean difference between the two samples to judge the degree of difference between the original thin section images and the generated thin section images. Unlike FID, KID applies the Gaussian kernel function to non-linearly map each feature vector to the kernel Hilbert space, to obtain feature representation information similar to human perception.

In the image sharpness detection experiment, CA-StyleGAN and similar image generation algorithms were trained. 1000 cast thin section images were generated by each algorithm. The IS, FID and KID values were calculated for each algorithm, the number of experiments was 50, and the IS, FID and KID values were the mean value of 50 times. By comparing algorithms, five similar image generation algorithms of StyleGAN^4^, CycleGAN^21^, DCGAN^22^, WGAN-GP^23^ and LS-GAN^24^ were selected.

#### Image distortion detection experiment design

The image distortion detection experiment uses the objective performance index Peak Signal to Noise Ratio (PSNR) and Multi-Scale Structural Similarity Index (MS-SSIM) as evaluation indexes^25^. PSNR determines the distortion degree of each component of the generated thin section image by calculating the pixel error of each component of the original thin section image and each component of the generated image. A higher score indicates a higher fidelity of the components in the generated thin section image. The PSNR evaluation method can be represented by Eq. (20).

20 $$PSNR = 10 \times \log_{10} \frac{{\left( {MAX_{I} } \right)^{2} }}{MSE} = 20 \times \log_{10} \frac{{MAX_{I} }}{{\sqrt {MSE} }}$$

where $MAX_{I}$ represents the maximum pixel in each component of the original thin section image. The image format in this section is uint8, and the maximum pixel is 255; MSE represents the mean squared error between each component I of the original thin section image and each component K of the generated thin section image, defined by Eq. (21). ${\text{m}} \times n$ represents the image size (In this section, the size of both the original image and the generated image is 512 pixels × 512 pixels).

21 $$MSE = \frac{1}{m \times n}\sum\limits_{{{\text{i}} = 0}}^{m - 1} {\sum\limits_{j = 0}^{n - 1} {\left[ {I\left( {i,j} \right) - K\left( {i,j} \right)} \right]} }^{2}$$

Since both the original thin section image and the generated thin section image are color images in this section, the PSNR of the RGB channels of each component in the thin section image was calculated separately, and the mean values were taken to reflect the fidelity of the components in the generated image.

MS-SSIM follows the same principle as SSIM. It accumulates the similarity score by comparing the similarity of luminance, resolution, contrast and related structures between the components of the original thin section image and the components of the generated image, and iterates the image down-sampling repeatedly to judge the similarity of the components of the generated image. The MS-SSIM evaluation method can be represented by Eq. (22).

22 $$MS - SSIM\left( {x,y} \right) = \left[ {l\left( {x,y} \right)} \right]^{\alpha M} \times \prod\limits_{j = 1}^{M} {\left[ {c\left( {x,y} \right)} \right]^{{\beta_{j} }} \times } \left[ {s\left( {x,y} \right)} \right]^{{\gamma_{j} }}$$

where x represents each component of the original thin section image; y represents each component of the generated thin section image; l represents luminance; c represents contrast; s represents structure; $\alpha M$, $\beta_{j}$ and $\gamma_{j}$ represent indices that adjust the relative importance of different components, $\alpha M$ represents the index of luminance l calculated only in the last iteration process; $l\left( {x,y} \right)$ represents the luminance comparison between each component of the original thin section image and each component of the generated thin section image; $c\left( {x,y} \right)$ represents the contrast comparison between each component of the original thin section image and each component of the generated thin section image; $s\left( {x,y} \right)$ represents the structure comparison between each component of the original thin section image and each component of the generated thin section image. $\alpha > 0,\beta > 0,\gamma > 0$. The calculation process of $l\left( {x,y} \right)$ can be represented by Eqs. (23), (24) and (25); the calculation process of $c\left( {x,y} \right)$ can be represented by Eqs. (26), (27) and (28); and the calculation process of $s\left( {x,y} \right)$ can be represented by Eq. (29).

23 $$l\left( {x,y} \right) = \frac{{2\mu_{x} \mu_{y} + c_{1} }}{{\mu_{x}^{2} + \mu_{y}^{2} + c_{1} }}$$

24 $$\mu_{x} = \frac{1}{H \times W}\sum\limits_{i = 1}^{H} {\sum\limits_{j = 1}^{W} {x\left( {i,j} \right)} }$$

25 $$\mu_{y} = \frac{1}{H \times W}\sum\limits_{i = 1}^{H} {\sum\limits_{j = 1}^{W} {y\left( {i,j} \right)} }$$

where $\mu_{x}$ represents the mean value of pixels of each component of the original thin section image; $\mu_{y}$ represents the mean value of pixels of each component of the generated thin section image; $c_{1} = \left( {{\text{k}}_{1} ,L} \right)^{2}$ is to prevent the denominator from being zero, ${\text{k}}_{1}$ is a constant, ${\text{k}}_{1} < < 1$, and the default value is 0.01; L represents the range of pixel values [0, 255].

26 $$c\left( {x,y} \right) = \frac{{2\sigma_{x} \sigma_{y} + c_{2} }}{{\sigma_{x}^{2} + \sigma_{y}^{2} + c_{2} }}s\left( {x,y} \right)$$

27 $$\sigma_{x} = \left[ {\frac{1}{H \times W - 1}\sum\limits_{i = 1}^{H} {\sum\limits_{j = 1}^{W} {x\left( {i,j} \right)} } - \mu_{x} } \right]^{1/2}$$

28 $$\sigma_{y} = \left[ {\frac{1}{H \times W - 1}\sum\limits_{i = 1}^{H} {\sum\limits_{j = 1}^{W} {x\left( {i,j} \right)} } - \mu_{y} } \right]^{1/2}$$

where $\sigma_{x}^{2}$ represents the variance of pixels of each component of the original thin section image; $\sigma_{y}^{2}$ represents the variance of pixels of each component of the generated thin section image; $c_{2} = \left( {{\text{k}}_{2} ,L} \right)^{2}$ is to prevent the denominator from being zero, ${\text{k}}_{2}$ is a constant, and the default value is 0.03; L represents the range of pixel values [0, 255].

29 $$s\left( {x,y} \right) = \frac{{\sigma_{xy} + c_{3} }}{{\sigma_{x} \sigma_{y} + c_{3} }}$$

where $\sigma_{xy}$ represents the covariance of pixels of each component of the original thin section image and pixels of each component of the generated thin section image; $c_{3}$ is a constant to prevent the denominator from being zero, and is usually taken as $c_{3} = c_{2} /2$.

In the experimental process, to simplify parameter selection, Eq. (22) was set as $\alpha_{j} = \beta_{j} = \gamma_{j}$, and the cross-scale was normalized to make different parameter settings comparable. The normalized cross-scale is shown in Eq. (30). Based on previous experimental results, the scale parameters are set as follows (Eq. 31).

30 $$\sum\limits_{j = 1}^{M} {\gamma_{j} = 1}$$

31 $$\left\{ \begin{gathered} \beta_{1} = \gamma_{1} = 0.0448 \hfill \\ \beta_{2} = \gamma_{2} = 0.2856 \hfill \\ \beta_{3} = \gamma_{3} = 0.3001 \hfill \\ \beta_{4} = \gamma_{4} = 0.2363 \hfill \\ \alpha_{5} = \beta_{5} = \gamma_{5} = 0.1333 \hfill \\ \end{gathered} \right.$$

In the image distortion detection experiment, by comparing CA-StyleGAN with contrast algorithms, 1000 cast thin section images were generated and screened for each algorithm, their respective PSNR and MS-SSIM values were calculated, and the number of experiments was 50 times. The final PSNR and MS-SSIM values were the mean value of 50 times. By comparing algorithms, five similar image generation algorithms of StyleGAN, CycleGAN, DCGAN, WGAN-GP and LS-GAN were selected.

#### Annotation accuracy detection experiment

The annotation accuracy detection experiment uses accuracy as the evaluation criterion. It compares the number of components and the corresponding number of pixels between SALM annotation and manual annotation, and builds a confusion matrix to analyze the accuracy of SALM in component annotation. The calculation of accuracy $AR$ can be represented by Eq. (32). Where, $L_{{{\text{num}}}}$ represents the number of components or pixel values correctly annotated by SALM, and $H_{num}$ represents the number of components or pixel values annotated by geological experts.

32 $$AR = \frac{{L_{{{\text{num}}}} }}{{H_{num} }} \times 100\%$$

In the annotation accuracy detection experiment, 50 thin section images were selected as experimental data, the SALM was applied for annotation, the number of correctly annotated pixels for each component in each image was counted, and the mean value of each component of 50 thin section images was calculated. The results were compared with those annotated by geological experts. The number of experiments was 10 times, and the result of the confusion matrix was the mean value of 10 experiments.

#### Annotation efficiency detection experiment

The annotation efficiency detection experiment takes time (in seconds) as the evaluation criterion to analyze the time required for SALM annotation under different datasets, assessing the annotation efficiency of SALM and its stability under different datasets. The number of annotated thin section images was 500, the number of experimental datasets was set to $N,N \in \left\{ {100,200,300,400,500} \right\}$, and the time required for SALM to annotate all images was analyzed. The number of experiments was 10 times, and the mean value of 10 experiments was finally calculated.

## Experimental effect analysis

### Analysis of image sharpness detection experiment effect

**Figure 5:**
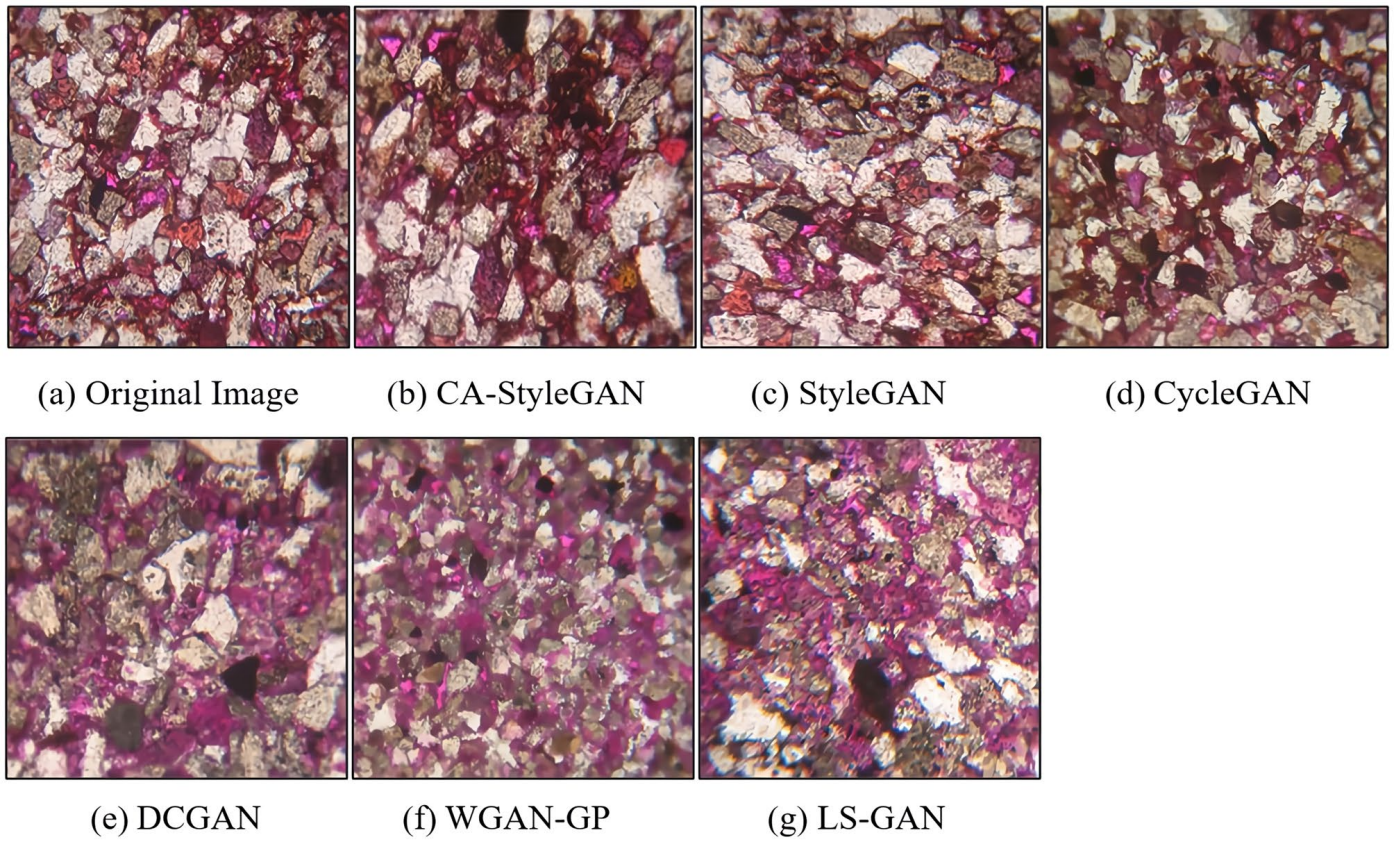
6 models generate image effects: (**a**) Original Image; (**b**) Image generated by CA-StyleGAN; (**c**) Image generated by StyleGAN; (**d**) Image generated by CycleGAN; (**e**) Image generated by DCGAN; (**f**) Image generated by WGAN-GP; (**g**) Image generated by LS-GAN.

According to the experimental design for image sharpness detection, experiments were conducted, the mean values of IS, FID and KID in 50 experiments were obtained, and the calculation results are shown in Table 2. The images generated by CA-StyleGAN, StyleGAN, CycleGAN, DCGAN, WGAN-GP and LS-GAN are depicted in Fig. 5. Due to performance limitations, the images generated by four models of CycleGAN, DCGAN, WGAN-GP and LS-GAN were adjusted to 256 pixels × 256 pixels.

**Table 2:** The calculation results of the evaluation index of image sharpness detection experiment.

Method/pixel	CA-StyleGAN 512 × 512	StyleGAN 512 × 512	CycleGAN 256 × 256	DCGAN 256 × 256	WGAN-GP 256 × 256	LS-GAN 256 × 256
FID	23.85	68.17	276.37	292.03	312.30	325
IS	3.02	2.23	2.19	1.24	1.15	1.07
KID	0.05	0.13	0.21	0.35	0.50	0.48

From Table 2, it can be observed that the CA-StyleGAN proposed in this paper achieved the highest IS value of 2.42 when generating thin section images, which was higher than the other five methods by 0.79, 0.83, 1.78, 1.87 and 1.95, indicating that the images generated by CA-StyleGAN exhibit good diversity and quality. The lowest FID score was 23.85, which was lower than 44.32, 252.52, 268.18, 288.45 and 301.15 of the other five methods, indicating that the images generated by CA-StyleGAN have the highest similarity to the original thin section image samples. The lowest KID value was 0.04, which was lower than 0.08, 0.16, 0.30, 0.45 and 0.43 of the other five methods, showing the minimal difference between the images generated by CA-StyleGAN and the original thin section images.

According to Fig. 5, the images generated by the CA-StyleGAN model are the closest to the real cast thin section images in terms of component arrangement, color features, component contours and edge boundaries. The images generated by the StyleGAN model exhibit clear color features and boundaries, but the arrangement of components is more complex and diverse, and there are distortion-like phenomena. The images generated by the CycleGAN model have blurred components and also exhibit distortion-like phenomena. The images generated by DCGAN, WGAN-GP and CycleGAN models not only feature diverse and complex component arrangement, distortion-like phenomena and blurred image components, but also display blurred component contours, uneven color tones and “component dissolution-like” phenomena.

### Analysis of image distortion detection experiment effect

Based on the image distortion detection experiment design, experiments were conducted, and the mean values of PSNR and MS-SSIM for each component of the thin section in 50 experiments were obtained, and the calculation results are shown in Tables 3 and 4.

**Table 3:** Calculation results of PSNR evaluation index of image distortion degree detection experiment.

Algorithm	PSDR/dB								
	Quartz	Feldspar	Lithic	PP	CP	CDP	Microcrack	Overall	Average
CA-StyleGAN	23.63	21.83	24.54	23.87	22.04	22.73	23.06	23.05	23.10
StyleGAN	14.53	12.04	15.34	14.42	11.78	13.76	14.25	13.63	13.73
CycleGAN	15.36	12.89	16.76	15.78	13.42	14.43	15.13	14.59	14.82
DCGAN	18.82	16.14	19.32	18.53	16.89	17.97	18.28	17.50	17.99
WGAN-GP	12.9	10.36	13.31	12.94	10.73	11.96	12.04	11.58	12.03
LS-GAN	11.4	9.29	12.39	11.75	9.42	10.72	10.98	10.34	10.85

**Table 4:** Calculation results of MS-SSIM evaluation index of image distortion degree detection experiment.

Algorithm	MS-SSIM/%								
	Quartz	Feldspar	Lithic	PP	CP	CDP	Microcrack	Overall	Average
CA-StyleGAN	97.33	96.34	97.29	96.52	95.56	95.89	96.38	96.34	96.47
StyleGAN	88.13	85.87	88.61	87.23	85.98	87.15	87.93	87.33	87.27
CycleGAN	88.64	86.02	89.1	88.28	86.35	87.96	88.17	87.84	87.79
DCGAN	92.5	90.33	92.68	91.85	90.03	91.97	92.02	91.79	91.63
WGAN-GP	83.85	80.95	84.2	83.55	81.41	83.09	83.15	82.69	82.89
LS-GAN	82.35	80.12	83.21	82.34	79.74	81.55	82.22	81.02	81.65

Table 3 shows that the CA-StyleGAN proposed in this paper achieved the highest PSNR value when generating thin section images, with all components above 21dB. The PSNR value of the entire image was closest to the mean value of PSNR of each component (with an error of 0.05dB), indicating that each component of thin section images generated by CA-StyleGAN has the highest fidelity.

From Table 4, it can be seen that the CA-StyleGAN proposed in this paper achieved the highest MS-SSIM value when generating thin section images, with all components above 95%. The PSNR value of the entire image was closest to the mean value of MS-SSIM of each component (with an error of 0.11%), indicating that each component of thin section images generated by CA-StyleGAN has the highest similarity to the original thin section images.

According to the PSNR and MS-SSIM values for each component, the values of Feldspar and CP are relatively low, which is mainly due to the similar optical and geometric characteristics of Feldspar and Quartz, Lithic, the similar optical and geometric characteristics of CP and PP, CDP. Additionally, by analyzing the difference in PSNR and MS-SSIM values between Feldspar and Quartz/Lithic as well as between CP and PP/CDP, the addition of category attention mechanism avoids the impact of the lower number of Feldspar and CP components on the training process of CA-StyleGAN (with the smallest difference in PSNR and MS-SSIM values between Feldspar and Quartz/Lithic as well as between CP and PP/CDP).

To sum up, the thin section images generated by CA-StyleGAN exhibit high fidelity.

### Analysis of annotation accuracy detection experiment effect

**Figure 6:**
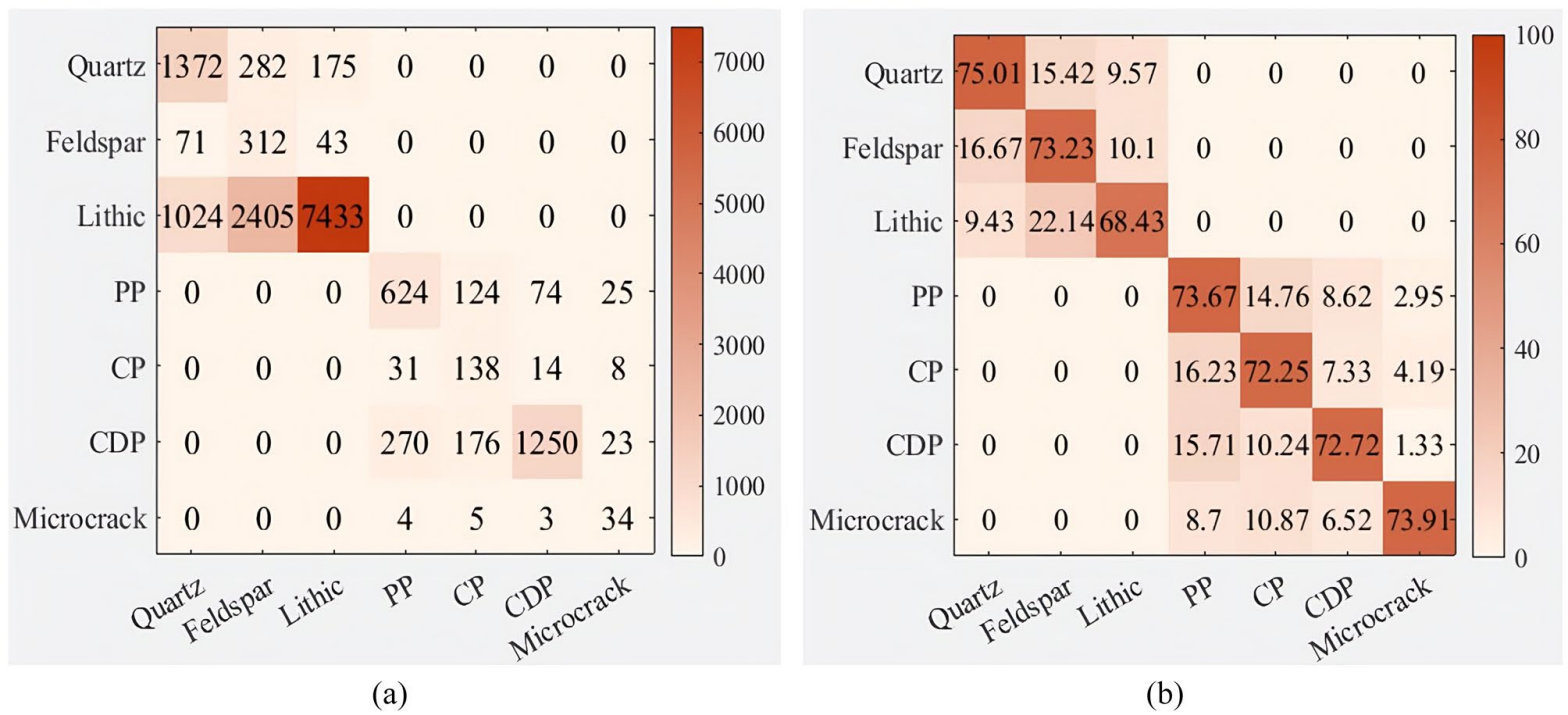
Quantity and accuracy of each component of thin section labeling: (**a**) Confusion matrix of the number of labels for each component; (**b**) Confusion matrix of each component labeling accuracy.

**Figure 7:**
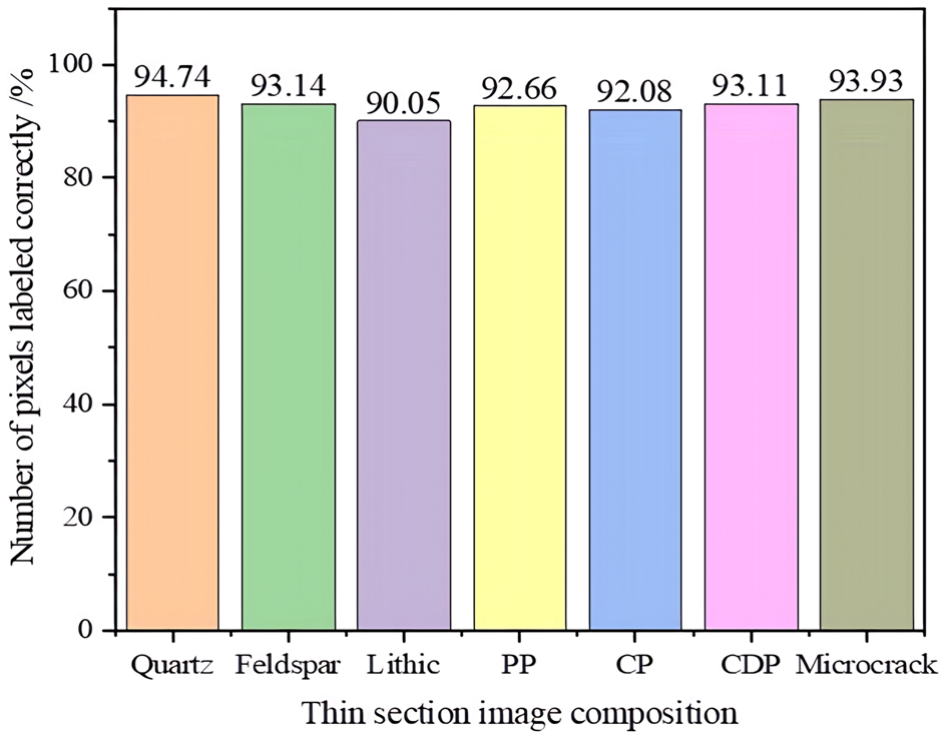
Accuracy of the number of pixels for each component of thin slice.

**Figure 8:**
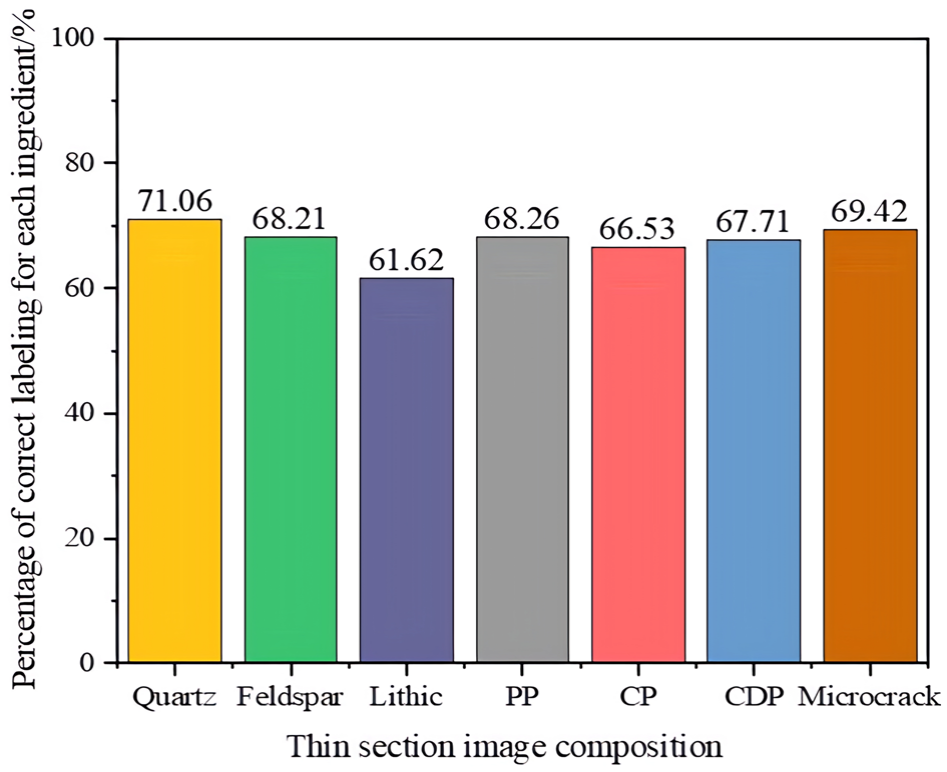
Accuracy of sheet component labeling.

**Figure 9:**
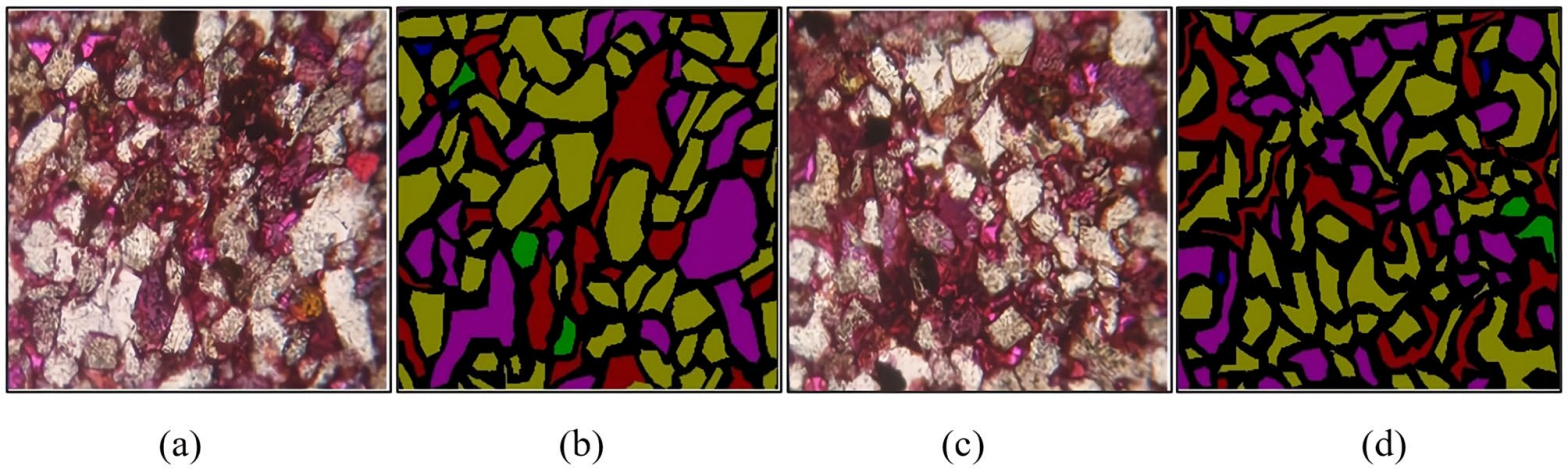
Generate images and their semantic labels: (**a**) Generating image1; (**b**) Semantic labeling 1; (**c**) Generating image 2; (**d**) Semantic labeling 2.

Based on the annotation accuracy detection experiment design, experiments were conducted to statistically analyze the average number of each component and their pixels in 10 SALM annotations, and the statistical results are shown in Table 5. The confusion matrix of the number of annotations and annotation accuracy for each component was established based on the annotation situation, as shown in Fig. 6 (The color bar represents the degree. The darker the color, the more the number of ingredients labeled and the higher the accuracy of labeling). The accuracy of the number of annotated pixels for each component is depicted in Fig. 7. The final annotation accuracy is shown in Fig. 8. Some annotated images are shown in Fig. 9, in which purple represents Quartz, green represents Feldspar, yellow represents Lithic, blue represents PP, and red represents CDP.

**Table 5:** SALM labels each component and counts the number of pixels.

	Quantity and proportion of each component of the original image	Generate the number and proportion of each component of the image	Correctly label the quantity of each ingredient	Number and proportion of pixels in each component of the original image	Generate the number and proportion of pixels of each component of the image	Correctly label the number of pixels of each component
Quartz	2176 (14.07%)	1829 (11.49%)	1372	3,310,625 (25.64%)	3,207,254 (24.47%)	3,038,582
Feldspar	447 (2.89%)	426 (2.68%)	312	723,542 (5.60%)	692,335 (5.28%)	644,823
Lithic	8405 (63.40%)	10,862 (68.22%)	7433	7,320,953 (56.71%)	7,624,569 (58.17%)	6,759,211
PP	768 (4.97%)	847 (5.32%)	624	378,645 (3.01%)	394,852 (3.01%)	365,868
CP	144 (0.93%)	191 (1.20%)	138	133,529 (1.12%)	146,878 (1.12%)	135,249
CDP	2091 (13.51%)	1719 (10.80%)	1250	991,202 (7.68%)	989,052 (7.55%)	920,952
Microcrack	35 (0.23%)	46 (0.29%)	34	51,534 (0.40%)	52,538 (0.40%)	49,347

According to Table 5, the proportion of the number of components and the number of pixels in the generated thin section images is roughly similar to that of the original images, with a proportion error of less than 5% within each component. This indicates that the proportion of each component in the generated thin section images accords with that in the original images, demonstrating a high coincidence degree.

From Fig. 6, it can be observed that SALM exhibits higher accuracy in annotating components such as Quartz, Feldspar, PP, CP and Microcrack, which have distinct color features and relatively large boundary color difference. The accuracy for these components is above 73%. However, the accuracy is relatively lower for complex components like Lithic and CDP, which is 68.43% and 72.25%. The main reason for this discrepancy is that the optical characteristics of Lithic component are similar to those of Quartz and Feldspar, and geological experts need to use two image features of thin sections under plane-polarized light and thin sections under cross-polarized light to annotate particles with less obvious features, leading to a relatively low accuracy of semi-automatic annotation by using plane-polarized light images. The particles of CDP component are completely dissolved to form pores, so some CDP component pixels have similar color features to other types of pores (especially PP pores), leading to a relatively higher mislabeling rate. Additionally, according to Table 5, the number and proportion of Microcrack component pixels are less, it has obvious color boundary (as can be proved in Fig. 9), so the accuracy of Microcrack labeling is relatively high (73.91%). The main reason for mislabeling may be that Microcrack passes through PP, CP and CDP. As a result, Microcrack is labeled as other pore components.

Figure 7 shows that SALM achieves high accuracy (> 88.65%) in labeling the pixel count of correctly annotated thin section components. However, the accuracy is relatively lower for Lithic and CDP components, mainly due to the complex component of Lithic, which makes its pixel features similar to Quartz and Feldspar, resulting in a relatively high mislabeling rate; The pores in CDP are mislabeled as PP and CP. Additionally, considering the annotation accuracy of each component in Fig. 6, it can be inferred that SALM is able to correctly annotate the components provided that the components of thin sections are correctly labeled. Thus, the main factor affecting SALM annotation is whether each component can be correctly identified.

Figure 8 indicates that the accuracy of SALM semi-automatic annotation for each component ranges between 61 and 72%, and it can replace geological experts in completing most thin section annotation tasks, thus possessing practical value.

### Analysis of annotation efficiency detection experiment effect

**Figure 10:**
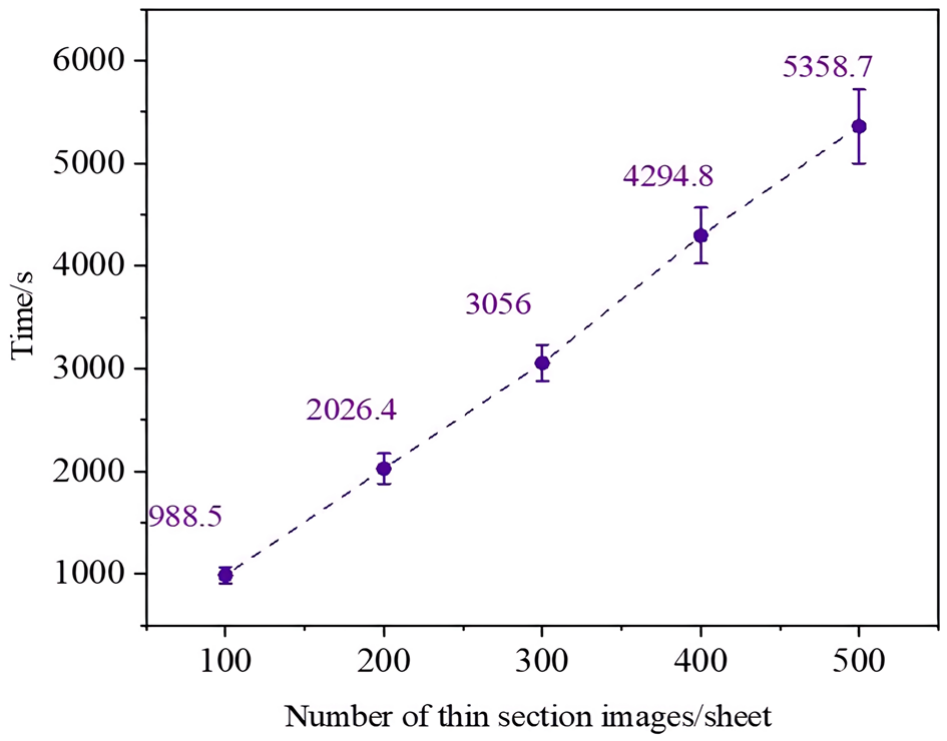
Labeling time for different number of thin sections.

Experiments were conducted based on the annotation efficiency detection experiment design to obtain the SALM annotation time under different experimental datasets of $N,N \in \left\{ {100,200,300,400,500} \right\}$ in 10 experiments, and the experimental results are shown in Fig. 10.

As can be seen from Fig. 10, the annotation time of SALM is positively correlated with the number of thin sections under different thin section datasets, indicating that the annotation time of SALM is less affected by dataset size. However, the error bar suggests that with an increase in thin section data volume, the annotation time may fluctuate within a certain range. This fluctuation is mainly caused by some thin section images having a larger number of components or more complex geometric shapes, leading to a slight increase in the required annotation time. Overall, the annotation time tends to be linearly related to the number of thin section images. Additionally, it is noted that the annotation efficiency of SALM is approximately 10 s per image, demonstrating a high annotation efficiency that significantly reduces the time required for thin section annotation.

## Conclusion

To address the scarcity of thin section images of tight oil reservoirs and insufficient datasets, we proposed a method based on SACA-StyleGAN to realize the generation and semi-automatic annotation of cast thin section images of tight oil reservoir. This method provides data support for intelligent identification of thin section components and reduces the time cost of thin section image labeling by geological experts, thereby improving thin section image labeling efficiency. Through theoretical exposition and experimental demonstration, the following research conclusions are drawn:

1. By comparing CA-StyleGAN with StyleGAN, CycleGAN, DCGAN, WGAN-GP and LS-GAN, it is found that thin section images generated by CA-StyleGAN exhibit high diversity and image quality with low distortion, indicating that thin section images generated by CA-StyleGAN are the most realistic compared to the original thin section images.
2. The accuracy of SALM annotation for each component ranges from 61 to 72%, and it can replace geological experts in completing most labeling tasks, demonstrating practicality. However, the accuracy still needs to be improved.
3. SALM demonstrates good stability and annotation efficiency under different datasets, with a labeling time of approximately 10 s per image.
4. In general, SACA-StyleGAN is suitable for generating and labeling cast thin section images of tight oil reservoir, and can provide high-quality datasets for intelligent identification of cast thin section images.

However, the SACA-StyleGAN method still fails to solve the problems such as the long training time of StyleGAN, the large number of parameters and the annotation accuracy to be improved. In future work, we plan to optimize the structure of the SACA-StyleGAN model to reduce the training time and the number of parameters, and improve the accuracy of SALM annotation and apply the SACA-StyleGAN method to the generation of different types of images of unconventional reservoirs, enhancing the scalability and applicability of the method.

## Data Availability

The datasets generated during and analyzed during the current study are available from the corresponding author on reasonable request.

## Abbreviations

GANs: Generative adversarial networks
StyleGAN: A style-based generator architecture for generative adversarial networks
CA-StyleGAN: A StyleGAN with an added category attention mechanism
CycleGAN: Cycle-consistent generative adversarial network
DCGAN: Deep convolution generative adversarial networks
WGAN-GP: Wasserstein GAN with gradient penalty
LS-GAN: Least squares GAN
PP: Primary pore
CP: Casting pore
CDP: Cemented dissolution pore
RNN: Recurrent neural network
LSTM: Long short-term memory network
TTUR: Two time-scale update rule
SALM: Semi-automatic labeling mechanism
IS: Inception score
FID: Frechet inception distance
KID: Kernel inception distance
PSNR: Peak signal to noise ratio
MS-SSIM: Multi-scale structural similarity index
